# Temporary transvenous pacing guided by the combined use of ultrasound and intracavitary electrocardiography: a feasible and safe technique

**DOI:** 10.1186/s13089-019-0122-y

**Published:** 2019-04-04

**Authors:** Pablo Blanco

**Affiliations:** Intensive Care Physician, Intensive Care Unit, Clínica Cruz Azul, 2651, 60 St., 7630 Necochea, Argentina

**Keywords:** Transthoracic echocardiography, Cardiac pacing, Ultrasonography, Electrocardiography, Point-of-care

## Abstract

Temporary transvenous pacing is a crucial procedure in emergency and critical care medicine. While fluoroscopy guidance is considered the gold-standard to place the temporary pacing electrode catheter (EC), its use is usually limited by equipment availability and time. By contrast, ultrasound (US) guidance is a useful alternative, since it is widely available at the bedside, does not emit ionizing radiation and provides optimal times to active pacing with fewer complications. However, many times, in spite of visualizing the EC in the right ventricle using US, the pacing capture and sensing are not optimal, and thus, having an easily applicable method for best assessing this issue is desirable, especially if it could be combined with US without difficulties. With the purpose of illustrating the points made previously, the combined US-intracavitary electrocardiography technique is described in detail in this paper.

**Electronic supplementary material:**

The online version of this article (10.1186/s13089-019-0122-y) contains supplementary material, which is available to authorized users.

## Introduction

Temporary transvenous pacing (TVP) is a lifesaving procedure which is mainly indicated in patients with symptomatic bradyarrhythmias as well as in patients with specific tachyarrhythmias (i.e., overdrive pacing) [1–4].

Temporary transvenous pacing consists in inserting a temporary pacing electrode catheter (EC) into the right ventricle and then applying an electric stimulus with the goal of restoring effective cardiac depolarization and heart contraction, resulting in the delivery of an adequate heart rate and cardiac output [3].

Several complications can result from this critical procedure such as failure to secure venous access, failure to place the lead correctly, sepsis, puncture of arteries, lungs or myocardium and life-threatening arrhythmias [5]. Giving these facts, a safe method to monitor the EC insertion is desirable [3].

The placement of the EC can be achieved in several ways, including a blind technique as well as a couple of guided techniques, such as intracavitary electrocardiography (ECG) [6, 7], ultrasonographic-guided insertion (US) [8–10] and fluoroscopy [9, 10]. Since the blind technique is neither safe nor effective most of the times, and considering that fluoroscopy is not usually available at the bedside or patients are commonly unstable to be transferred to the radiology department, ECG and/or real-time ultrasonographic (US) guidance are generally chosen to assist in the procedure at the patient’s bedside. The combination of guided techniques for the placement of the EC is a valid and useful strategy, with the intention of making the procedure easy, safe, and effective. This is the case of the combination of US guidance with intracavitary ECG, which is easy to perform, may lead to a reduction in the time to active pacing and may avoid complications.

Regarding the selection of the route of insertion, this may be guided by several factors, such as the presence of hypovolemia, anticoagulation status, or adequate anatomy. As a general rule, the right-sided veins are preferred over the left because permanent systems are usually inserted on the left side and because it is often technically easier from the right side [5]. In general, the right internal jugular vein provides the most direct route to the right ventricle and it is associated with lowest rate of loss of ventricular capture and thus is the recommended route for using in practice [5]. Right subclavian/axillary route follows the right IJV and is preferred in patients with hypovolemia, given the ability of these vessels to remain patent even in patients with volume depletion. Femoral access can be performed with ease; however, it can be more difficult to advance the electrodes to the right ventricle, limits patient mobility, has a higher risk of venous thromboembolism, and offers the least stable wire position [11]. Of note, securing a venous access is not a minor issue, with a high failure rate reported among studies (average 15%, range 6–40%) [5]. Ultrasound-guided insertion of the introducer sheath, which is in fact a central venous canulation, has proven to improve canulation success and reduce complications related to the procedure [12]. Thus, all introducer sheaths should be placed under ultrasound guidance, unless there is no time to prepare the ultrasound equipment, such as in extreme situations (e.g., cardiopulmonary resuscitation), or eventually when ultrasound cannot be used for technical reasons (e.g., subcutaneous emphysema).

The combined technique (ultrasound and intracavitary ECG) will be described after discussing some aspects of both techniques when used in isolation.

## Feasibility of ultrasound guidance in placing the electrocatheter

Aguilera et al. [8] reported the transthoracic echocardiography (TTE)-guided insertion of the EC in nine patients in the ED. The EC was successfully observed by TTE in eight patients, allowing also to detect mispositioning of the EC and its adequate repositioning in three patients. Right IJV was used in six patients, while right subclavian vein was used in three. US-guided insertion of the introducer was not reported. The total time to pacemaker insertion was 4–13 min in this study.

The study carried out by Pinneri et al. [9] compared the placement of EC using TTE guidance (*n* = 53, with EC inserted through the internal jugular vein) versus fluoroscopy (*n* = 53, with EC inserted through the femoral vein). Although some of the differences between the two groups may be accounted for by the site of insertion and the guidance method, in the presence of an adequate acoustic window, the TTE guidance allows for a reliable temporary pacing with short procedural times, showing lower costs, avoiding the use of ionizing radiation and adding the advantage to perform the procedure at the bedside.

More recently, Ferri et al. [10] compared the insertion of the EC via the echo-guided approach (jugular vein) in 113 patients vs fluoroscopy (*n* = 90) via the femoral vein. In this study, the echo-guided approach reduced the median time to active pacing (22 vs 43 min), showed less complications (lower infections rates and puncture-related hematomas) and reduced the time to implantation of the definitive pacemaker.

In cases of inadequate cardiac windows, the use of transesophageal echocardiography (TEE) guidance has been anecdotally described [13]; however, this technique is invasive and it is not yet widely available in the emergency and critical care settings.

## Intracavitary ECG-guidance in placing the EC

While this technique is well-described in the literature, it is scarcely utilized in many EDs and ICUs. Although time to active pacing using this technique is not described in literature according to author´s knowledge, in practice, this is a reliable method to safely insert the EC in the RV and ensure a correct capture and sensing of the device [6, 7]. Although expected ECG patterns are observed based on the EC position, it is sometimes difficult to know where the EC is actually placed. For example, when it is placed in the coronary sinus, inferior vena cava or in RV perforations. Also, recording the intracavitary ECG as the EC is advanced is time-consuming in practice and not the best option when pacing urges, so having in practice a direct visualization of the EC passing through the tricuspid valve into the RV may save the time spent in recording these lead positions.

## Simultaneous use of ultrasound and intracavitary ECG: the technique

**Fig. 1:**
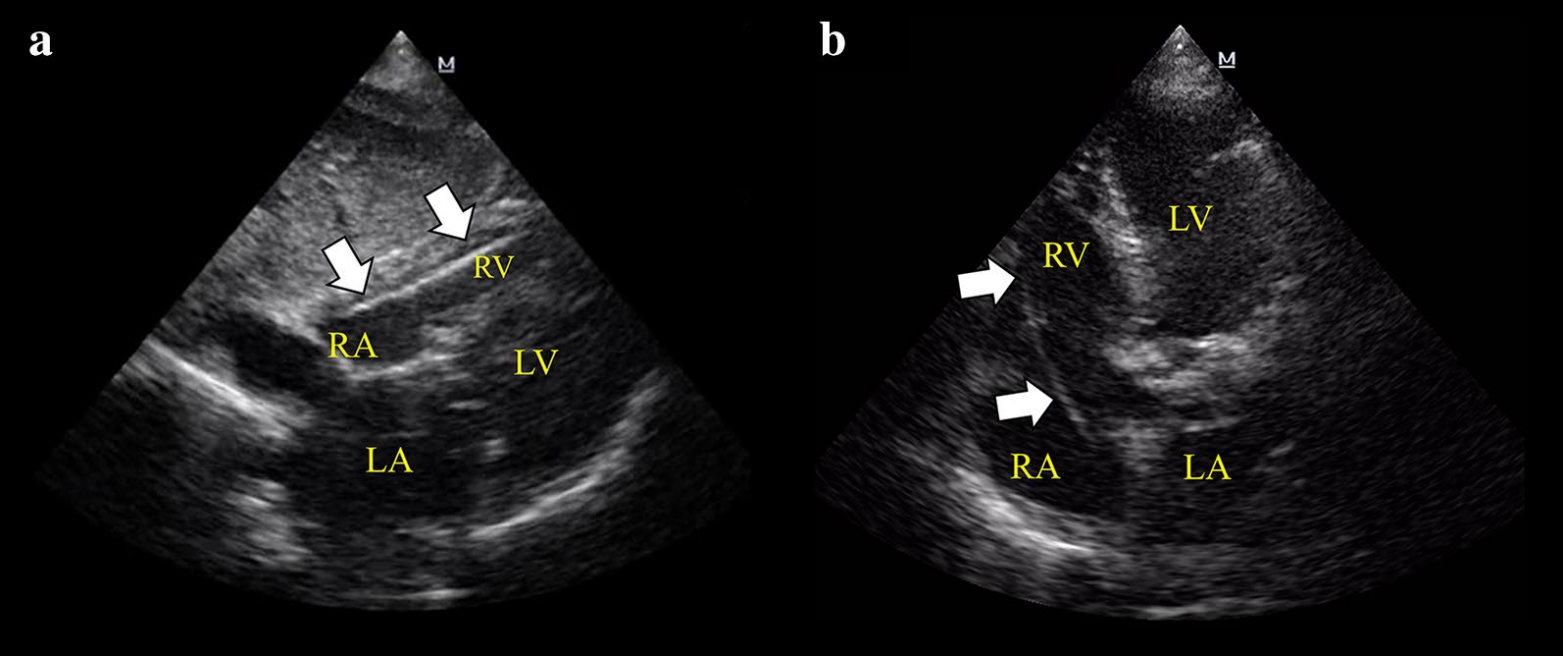
Subcostal 4-chamber view (**a**) and apical 4-chamber view (**b**) demonstrating the temporary pacing electrode catheter (EC, arrows) into the right ventricle (RV). *RV* right ventricle, *RA* right atrium, *LV* left ventricle, *LA* left atrium

**Fig. 2:**
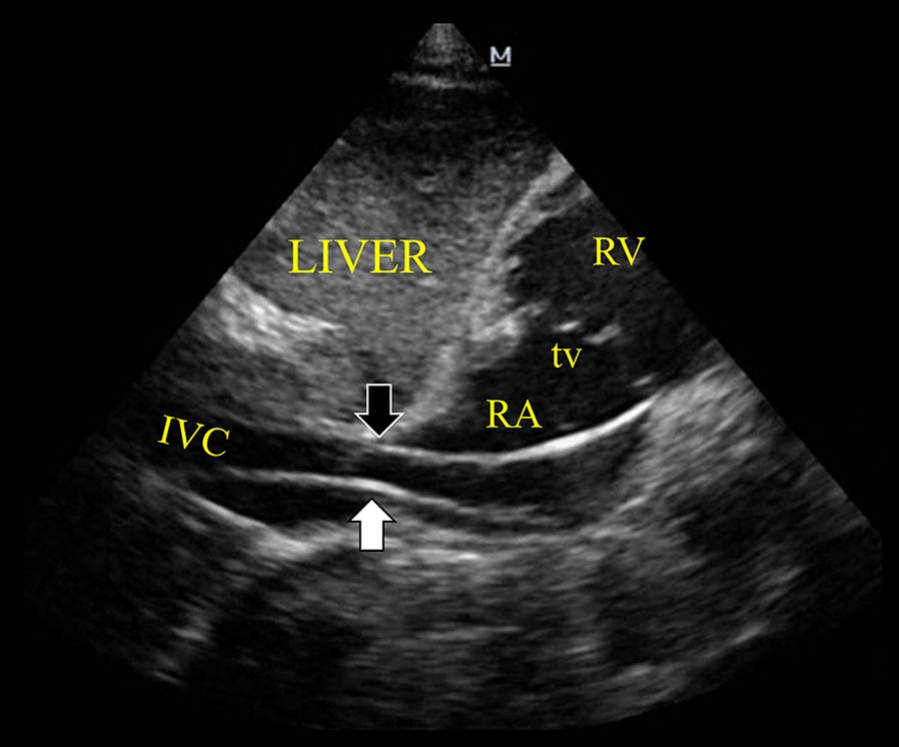
Subcostal inferior vena cava (IVC) view demonstrating the EC entering (white arrow) and getting out (black arrow) the IVC in direction of the right heart chambers. *RA* right atrium, *tv* tricuspid valve, *RV* right ventricle

After inserting the introducer sheath into the internal jugular (right internal jugular vein preferred as noted above) or subclavian/axillary vein both under US guidance, a 5-7F-bipolar EC (balloon-tipped floating catheter is preferred) is advanced by an operator while a second operator performs the transthoracic echocardiogram (TTE) exam. The EC is seen as a bright linear hyperechoic structure. The most important echocardiographic views to observe the EC are the subcostal four-chamber and the apical four-chamber, which are normally required to monitor the position of the EC while it is advanced into the right ventricle (RV). Both views allow practitioners to follow the EC into the right atrium (RA) and its progression through the tricuspid valve (TV) up to the preferred lead position into the RV apex (Fig. 1 and Additional file 1: Videos S1, Additional file 2: Videos S2). Other alternative EC positions, although somewhat unstable, are the interventricular septum, RV free wall, and RV outflow tract (RVOT). The inferior vena cava (IVC) view should be checked when, despite advancement of the EC, there is no apparent EC progression into the RV (Fig. 2 and Additional file 1: Videos S3). Due to the anatomic continuity between the superior vena cava and IVC, the EC inadvertently passing into the IVC is the most frequent incorrect placement observed in practice. Short-axis views (subcostal and/or parasternal) at the level of the great vessels are useful to detect the EC passing through the TV and a RVOT and, also importantly, to exclude an accidental misplacement into the pulmonary artery.

**Fig. 3:**
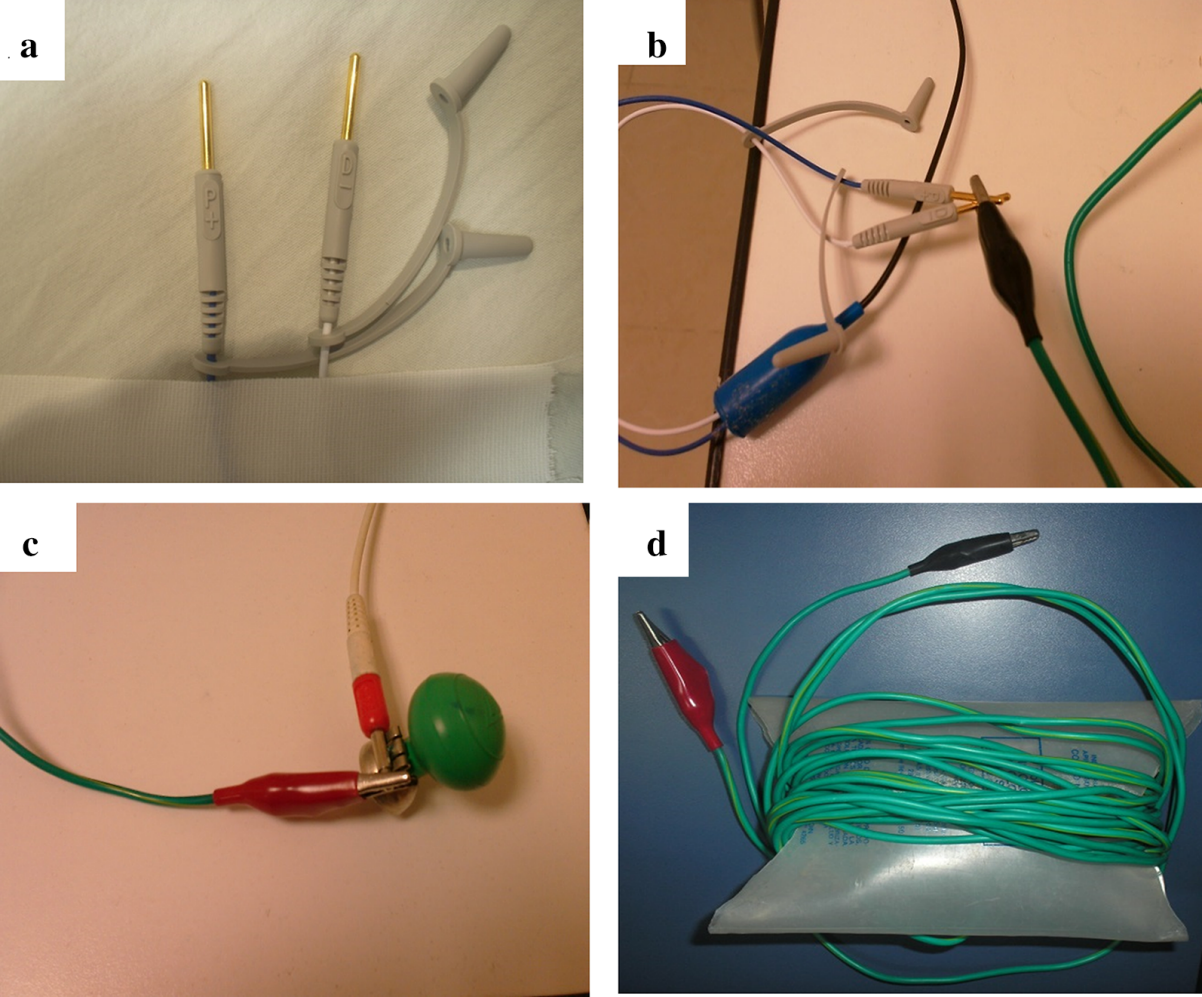
Intracavitary electrocardiogram (ECG) technique attaching the electrodes of the EC (**a**, **b**) to a precordial lead (V1 in this case, **c** by a cable with an alligator clip on each end (**d**). Of note, while the alligator clip is attached to a precordial lead as seen by its suction cup, this is in fact registering the cardiac electrical activity from an intracavitary view, thus it is indistinct whether or not it is placed on the patient’s chest; ECG limb leads are placed in the usual fashion

**Fig. 4:**
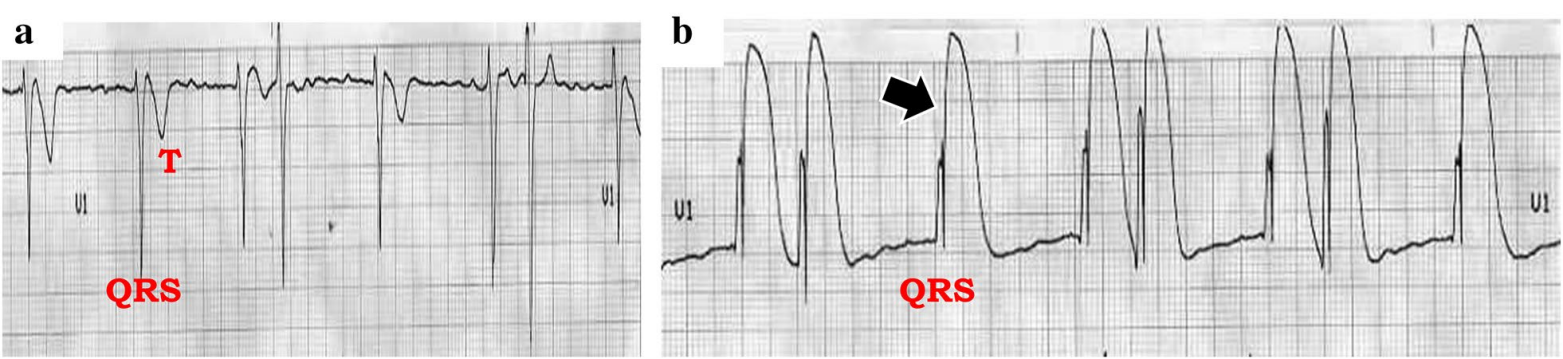
Intracavitary ECG (lead V1) showing the cardiac electrical activity when the EC is free-floating into the RV (**a**) and when this is contacting the endocardium of the RV (**b**), as demonstrated by a subepicardial injury pattern (arrow)

Although the lead is positioned into the RV by TTE, ultrasound usually does not provide complete certainty as to whether the tip of the EC is free-floating or in close contact with the endocardium of the RV wall, which is a critical requirement for the device to achieve an appropriate sensing and capture. One study showed that only 62% of the ECs seen in the RV have an adequate capture [8]. To assess this issue, an intracavitary ECG (Figs. 3 and 4 and Additional file 1: Videos S1) is then performed at this moment. To do so, a cable with one alligator clip (also called a “crocodile clip” outside of the United States) on each end is needed: one alligator clip is connected to the negative or distal pole from the end of the EC; the second alligator clip is attached to any of the precordial leads on the ECG machine; ECG limb leads are connected to the patient in the usual fashion [7]. Intracavitary ECG (recording the V lead that has the alligator clip attached) may allow to recognize the position of the EC as it is advanced from the superior vena cava up to the desired heart position. To demonstrate that the EC is in contact with the RV endocardium and not free-floating, an intracavitary ECG must show a subepicardial injury pattern, which is usually obtained at a lead depth of approximately 35–40 cm. If this injury pattern is not evident, minimal adjustments of the EC (backing off or advancing it a few centimeters) generally allow to achieve it without difficulty. Further EC advancement beyond 40–45 cm may result in coiling and also atypical lead positions instead of showing a RV subepicardial injury pattern [4, 5]. As a rule, the definite and optimal lead position is into the RV, with the demonstration of a clear IVC and pulmonary artery [4].

After a subepicardial injury is demonstrated on intracavitary ECG and the lead is connected to the generator which is now turned on, ventricular capture is normally achieved at low stimulation thresholds. Surface ECG will ordinarily show the pacemaker spikes accompanied by broad QRS complexes of left bundle branch block (LBBB) morphology. Sensing will also be optimal at low thresholds. A “benign” captured right bundle branch pattern (RBBB), although rare, can also be recorded in some patients [14]. While RBBB is observed in left-sided catheters (perforations) as well as in coronary sinus (CS) insertion, TTE confirmation of the EC positioned into the RV is reassuring [15, 16].

**Fig. 5:**
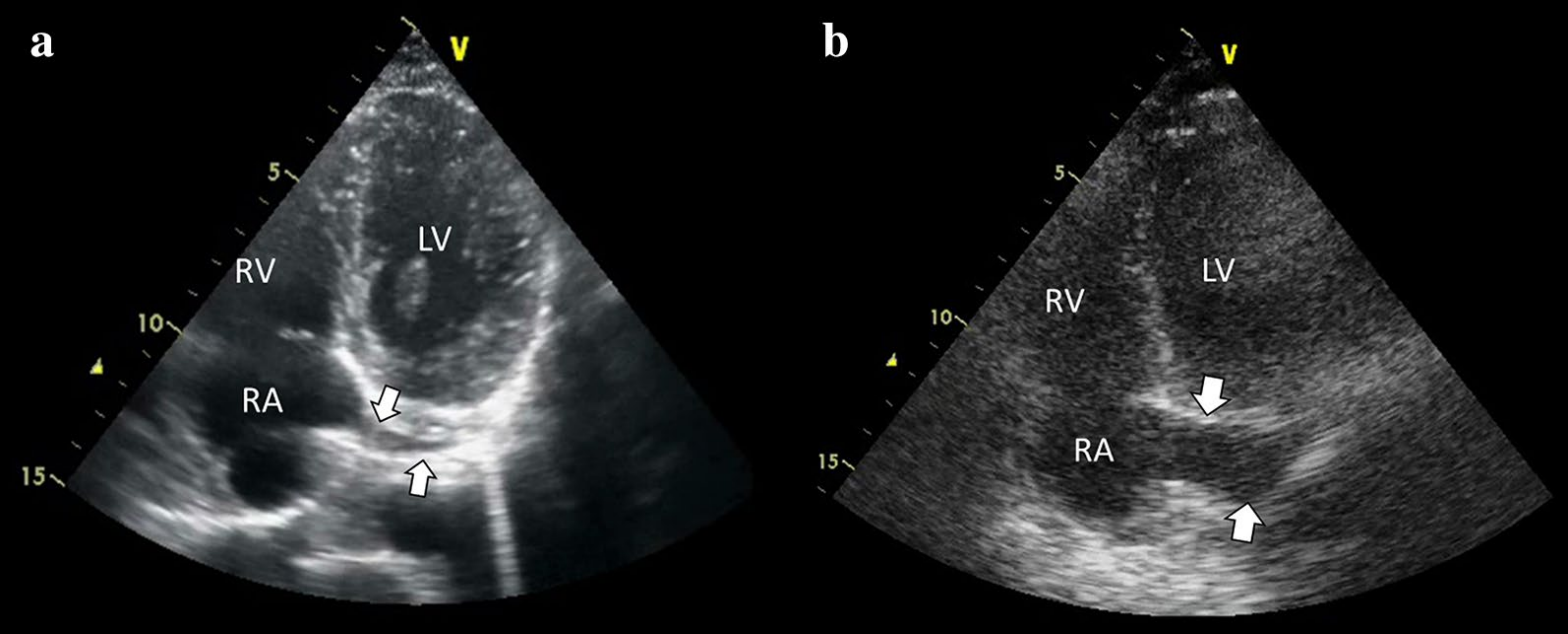
Coronary sinus view, obtained from a foreshortened apical 4-chamber view. **a** Normal coronary sinus (arrow). **b** Dilated coronary sinus (arrows)

Through the observation of the lead advancement by TTE, serious complications can be detected, such as septal perforations (interauricular and interventricular), RV free wall perforation and pericardial effusion, and eventually cardiac tamponade [16]. Misplacement into the pulmonary artery and IVC can also be readily detected by TTE [4, 8], allowing the redirection of the EC to an optimal position. The insertion in the coronary sinus (CS) [17] is another rare possibility, especially occurring when the CS is dilated or when a left-sided insertion (left IJV or subclavian vein) is attempted, and a persistent left superior vena cava is present. CS can be well-visualized in a foreshortened apical four-chamber view (Fig. 5) and, as previously mentioned, the captured QRS complexes will have a RBBB pattern. A lateral chest X-ray may aid in confirming a lead positioned in the CS. Of note, a CS lead placement allows for a more physiologic ventricular depolarization, with lesser degrees of inter- and intra-ventricular asynchrony in comparison with a RV position. Thus, there are lower risks of cardiac failure after TVP. In the absence of other complications or TVP malfunctioning, a CS lead can be safely kept in place [17]. The same principle of more physiologic ventricular depolarization applies also to RV leads that are not located in the ventricular apex, with lesser degrees of asynchrony in leads placed in the interventricular septum in comparison with apical leads. Unless the TVP is malfunctioning, these ECs do not usually require repositioning.

Once the capture and sensing of the device is adequate as demonstrated on the surface ECG, lung ultrasound is performed to rule in or out a pneumothorax (the presence or absence of lung sliding, along other signs of pneumothorax).

**Fig. 6:**
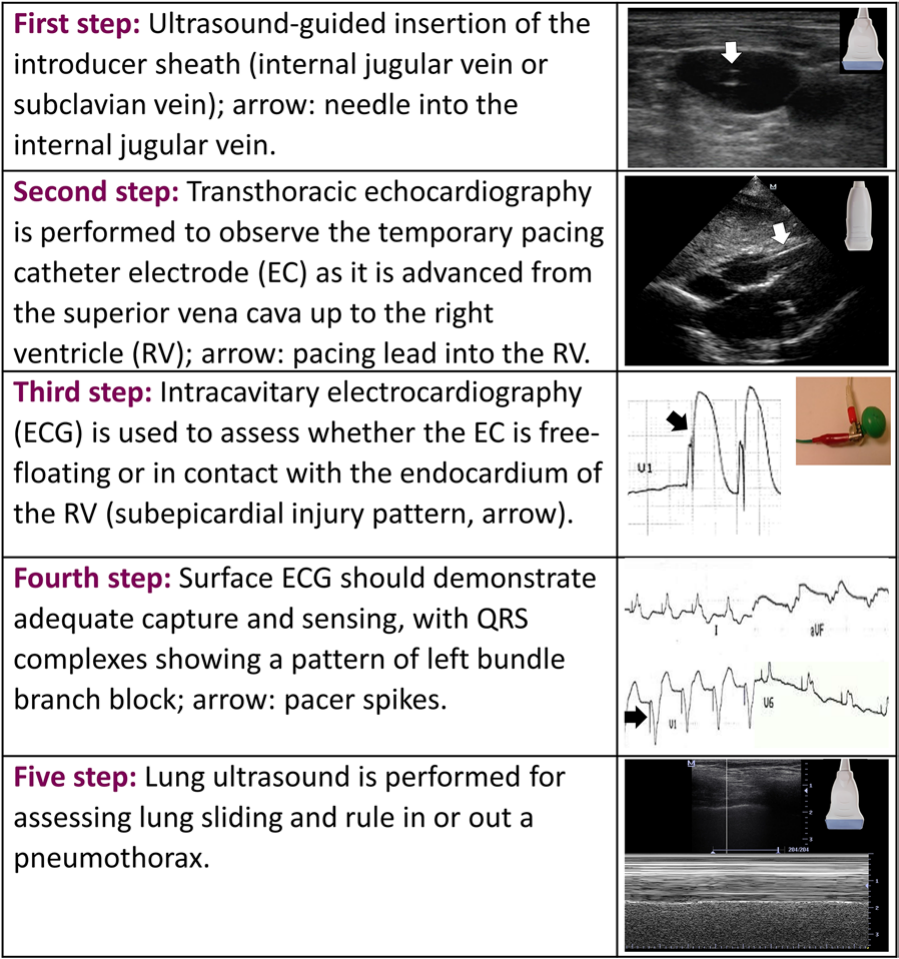
Summary of the combined technique: ultrasound and intracavitary ECG technique for temporary transvenous pacing

The complete technique is summarized in Fig. 6.

## Analysis of ultrasound plus intracavitary ECG-guidance to insert the EC

The combined use of US and intracavitary ECG to insert the EC appears as a potentially useful method for a secure lead placement in emergency settings. There are several benefits associated with this combined technique that need to be recognized. In the first place, the insertion of the introducer sheath under US guidance, which is associated with an improvement in first pass success and fewer complications in comparison with the landmark technique. Second, TTE may allow practitioners to quickly pass the EC to the RV, under direct visualization, thus allowing for the detection of complications or misplacements and the reposition of the EC in real time. Third, for intracavitary ECG, a rapid assessment of RV subepicardial injury pattern is achieved when the EC is in the RV, avoiding failed pacing captures and sensing. Finally, lung US is useful to rule in or out a pneumothorax in seconds, at the patient’s bedside.

Limitations of ultrasound and intracavitary ECG are the same as the ones established for each method when used in isolation.

Adding ECG-guidance to TTE in this way is easy, does not seem to add unusual complexity or extra time spent on the procedure and may actually improve the times to active pacing, avoiding complications.

## Limitations

This short communication is based on author’s years of experience performing this procedure in this way, achieving excellent results. However, prospective studies are needed to validate this simultaneous technique in large populations.

## Conclusions

While ultrasound is a feasible guidance to insert and monitor the EC placement at the patient’s bedside, the addition of intracavitary ECG allows for a better determination of RV wall lead-contacting, enhancing a correct pacemaker capture and sensing, without adding unusual complexity or spending extra time in the procedure. Emergency and critical care physicians must be aware of the benefits of using this combined method when transvenous pacing is needed.

## Additional files

**Additional file 1: Video S1.** Subcostal 4-chamber view showing in real time the entering of the temporary pacing electrode catheter (EC) into the RV, the intracavitary ECG when the EC is free-floating and when is contacting the endocardium, and the moment when the capture is achieved after connecting the electrodes of the EC to the generator device switched on.

**Additional file 2: Video S2.** Subcostal 4-chamber and apical 4-chamber view showing the pacing lead in the apex of the right ventricle, corresponding to Fig. 1.

**Additional file 3: Video S3.** Subcostal inferior vena cava (IVC) view demonstrating the pacing lead entering and getting out the IVC in direction of the right heart chambers, corresponding to Fig. 2.
